# The root's hidden ally: How the rhizosheath microbiome fortifies crops against drought

**DOI:** 10.1016/j.abiote.2025.100015

**Published:** 2025-12-07

**Authors:** Hassan Etesami, Amir Hosein Yadegari, Umarov Otabek, Bafayeva Zahro, Nafetdinov Shavakatullo, Avezov Tolqinjon

**Affiliations:** a Department of Soil Science, University of Tehran, Tehran 11369, Iran; b Department of Agronomy and Soil Science, Bukhara State University, Bukhara 200100, Uzbekistan; c Biology Department, Bukhara State University, Bukhara 200100, Uzbekistan

**Keywords:** Rhizosheath microbiome, Drought stress alleviation, Plant–microbe interactions, Sustainable agriculture, Climate resilience

## Abstract

Drought stress poses a significant threat to global agriculture, necessitating innovative strategies to enhance plant resilience. This review highlights the rhizosheath—the soil layer tightly bound to roots by mucilage and microbial biofilms—as a critical but underexplored microbial niche for sustainable drought mitigation. Unlike the vulnerable rhizosphere, the rhizosheath has a cohesive structure that acts as a protective “mini-oasis,” preserving moisture and sustaining microbial activity when water is scarce. We synthesize evidence showing that resident rhizosheath bacteria, including genera such as *Bacillus*, *Pseudomonas*, and *Azospirillum*, enhance plant drought tolerance through multiple mechanisms: improving soil structure and water retention, modulating phytohormone levels, facilitating nutrient acquisition, and activating antioxidant and genetic defense pathways in the plant. Despite promising laboratory findings, there has been little field-scale validation of these effects. Here, we critically assess translational challenges and outline future research priorities, such as understanding plant–microbe specificity and optimizing synthetic microbial consortia. Addressing these questions will enable manipulation of the rhizosheath microbiome for development of climate-resilient crops and securing food production in water-limited environments.

## 1 Introduction

Drought stress is a significant threat to global food security, and its frequency and severity are amplified by climate change [1,2]. The success of traditional mitigation strategies, such as breeding or genetic engineering of drought-tolerant cultivars, is often limited by lengthy development times, genetic complexity, and neglect of the soil ecological context, particularly the presence of beneficial root-associated microbial communities [3,4]. In recent years, manipulation of the plant microbiome has emerged as a novel tool for sustainable enhancement of crop resilience. Most attention has been focused on the rhizosphere—the soil zone influenced by root exudates [5]. However, the benefits of rhizosphere microbes can be severely limited under drought stress owing to desiccation and reduced microbial activity [[6], [7], [8]] (Table 1). This critical limitation underscores the need to identify microbial niches that remain functionally robust under water scarcity. This is where the rhizosheath becomes pivotal.

**Table 1:** Effects of drought stress on soil bacteria and their adaptive mechanisms.

Drought stress effects	Details	References
Osmotic stress	Water leaves the cell, causing dehydration and shrinkage	[9]
Free radical accumulation	Conformational protein changes, restricted enzyme efficiency, and electron transport chain issues	[10,11]
Nucleic acid damage	Chemical modifications (alkylation, oxidation), cross-linking, or base removal	[12,13]
Resource competition	Limited nutrients and water availability in the soil	[9,14]
Protein denaturation	Free radicals induce protein misfolding and denaturation	[13]
Lipid Peroxidation	Free radicals damage cell membranes, leading to cell lysis	[13]

Bacterial adaptations		

Compatible solutes	Accumulation of proline, glycine betaine, and trehalose to balance osmotic pressure	[9,11,15,16]
Heat shock proteins (HSPs)	Bind to misfolded proteins to prevent denaturation	[17,18]
Exopolysaccharides (EPS)	Production of EPS to protect the cell and its local environment	[19]
Spore formation	Formation of spores to survive extreme conditions	[16,20]
Ribosome storage	High quantities of ribosomes stored for rapid protein synthesis post-stress	[21]
Membrane adaptations	Changes in phospholipid fatty acid composition to maintain membrane integrity	[11,22]
Resource re-allocation	Increased efficiency of resource use within microbial cells	[23]

**Fig. 1:**
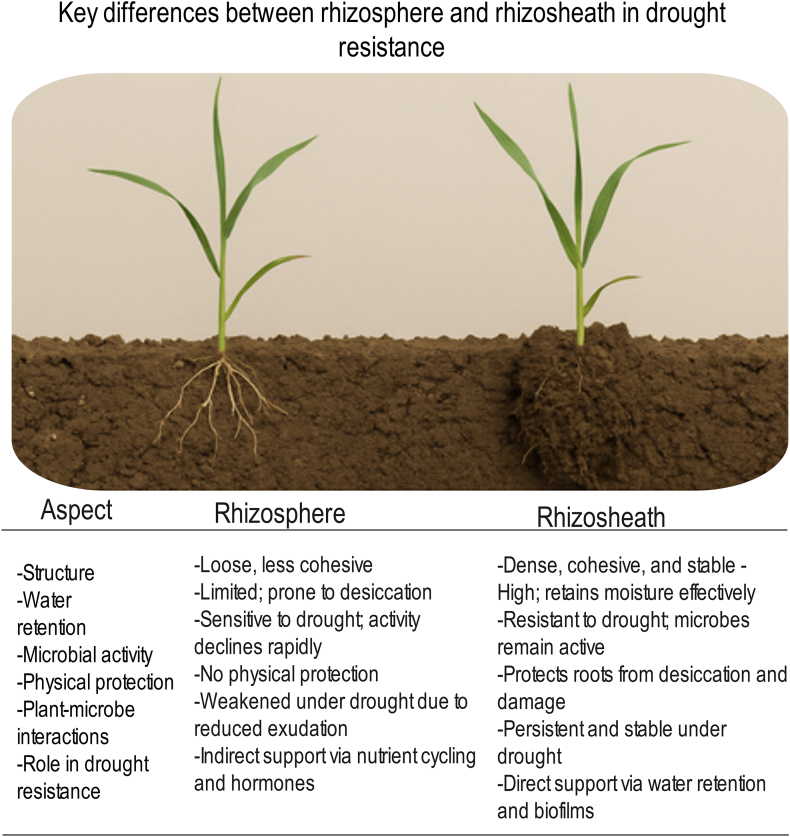
Key differences between the rhizosphere and the rhizosheath in drought resistance.

The rhizosheath—a specialized structure in which soil particles are tightly bound to roots by root mucilage and microbial biofilms—is a distinct and underexplored microenvironment [24,25] (Fig. 1). Unlike the more diffuse rhizosphere, the rhizosheath has a cohesive architecture and acts as a protective sheath, enhancing moisture retention, stabilizing soil–root contact, and maintaining a hydrated habitat for microbial life, even under severe drought [26,27]. The rhizosheath therefore serves as a natural reservoir for drought-adapted plant-growth-promoting bacteria (PGPBs). These bacteria, including genera such as *Bacillus*, *Pseudomonas*, and *Azospirillum*, can enhance plant drought tolerance through multiple mechanisms. These include the production of exopolysaccharides (EPSs) for soil aggregation; modulation of phytohormones to optimize root architecture and stomatal closure; synthesis of osmoprotectants; and activation of plant antioxidant defenses [28,29].

Despite the importance of its microbiome, the rhizosheath has received far less research attention than the rhizosphere. Many reviews have addressed the effects of general rhizosphere bacteria on plant drought tolerance, but the unique properties and microbial dynamics of the rhizosheath have rarely been summarized, limiting our ability to harness these specialized microbes for agricultural applications. In the present review, we synthesize current knowledge and highlight the transformative potential of rhizosheath-associated bacteria for the enhancement of crop drought resilience. We describe the unique structure and function of the rhizosheath, detail the diversity of its resident bacteria, and discuss their mechanisms of action. We then critically assess the challenges of translating laboratory findings into field-scale applications and outline future research directions focused on the development of effective, context-specific microbial inoculants. By addressing critical knowledge gaps, this review seeks to position the rhizosheath microbiome as a cornerstone of next-generation strategies for sustainable agriculture in water-limited environments.

## 2 A multifaceted approach: Existing strategies for mitigation of drought stress in crops

Addressing the pervasive challenge of drought requires an integrated approach that operates across multiple biological and agronomic levels. Whereas traditional methods have primarily focused on plant genetics and physiology, contemporary strategies encompass a broader spectrum of approaches, from molecular interventions to field-scale management practices. A systematic overview of these strategies, together with their advantages and limitations (Table 2). Genetic and molecular strategies include both conventional breeding and modern biotechnological tools such as marker-assisted selection and genetic engineering, aimed at the development of drought-tolerant cultivars. Key targets include genes responsible for the synthesis of osmoprotectants (e.g., proline), as well as those encoding stress-responsive transcription factors (e.g., DREBs) and aquaporins [[30], [31], [32], [33], [34]]. Physiological and organ-level interventions focus on the management of plant water relations and include practices such as regulated deficit irrigation to optimize water use and the application of anti-transpirants to reduce stomatal water loss [[35], [36], [37]]. Agronomic management practices include field-level strategies such as conservation tillage to minimize soil water evaporation, mulching to conserve soil moisture, and crop rotation with drought-adapted species to enhance system resilience [[38], [39], [40]]. Strategies based on plant–microbe interactions manipulate the plant's microbiome to enhance stress tolerance, using plant growth-promoting rhizobacteria (PGPRs) and arbuscular mycorrhizal fungi (AMF) to improve water and nutrient uptake, modulate phytohormone levels, and induce systemic tolerance [2,41]. Although all of these strategies can contribute to improving drought resilience, they are not without constraints. Genetic approaches can be time-consuming and may encounter regulatory and public-acceptance hurdles, and agronomic practices may be constrained by resource availability and specific environmental conditions [42,43]. Critically, many of these strategies are used in isolation, overlooking the dynamic interplay between the plant and its associated soil microbiome. Most research on plant–microbe interaction strategies has historically focused on the general rhizosphere, a zone that is itself highly vulnerable to desiccation. This review posits that the next frontier in sustainable drought mitigation may lie in harnessing specific, resilient microbial niches—most notably the rhizosheath.

**Table 2:** Strategies for mitigating drought stress in crops at multiple levels.

Level	Strategy	Description	Key advantages	Key limitations	Reference
Molecular	Genetic engineering	Direct manipulation of genes to enhance expression of drought-responsive proteins (e.g., LEA proteins, osmoprotectants, and transcription factors)	High precision; potential for introducing novel traits not present in the gene pool	Lengthy development time; significant regulatory hurdles and public acceptance issues (GMO); high cost	[43,44]
	Marker-assisted breeding	Using molecular markers to rapidly select for desirable drought-tolerant quantitative trait loci (QTLs) during conventional breeding	Faster than conventional breeding; avoids GMO regulations	Requires prior identification of reliable markers/QTLs; effectiveness is limited by existing genetic diversity	[45,46]
Organ-level	Root system architecture (RSA) modification	Breeding for traits like deeper roots, increased root hair density, and lateral root growth to maximize water foraging	Directly improves water access from deeper soil layers; based on natural genetic variation	Can be influenced by soil compaction; may involve trade-offs with energy allocation to shoot biomass	[[47], [48], [49]]
	Stomatal regulation	Selecting for genotypes with optimized stomatal density and responsiveness to close under water deficit	Reduces water loss through transpiration; relatively rapid response	Can limit CO_2_ uptake and photosynthesis, potentially reducing yield	[50,51]
Physiological	Osmotic adjustment	Breeding or managing plants to accumulate compatible solutes (e.g., proline and glycine betaine) to maintain cell turgor	Maintains cellular integrity and function under low water potential	Energy-costly process; high solute accumulation can sometimes be toxic	[[52], [53], [54]]
	Antioxidant defense system enhancement	Boosting the activity of enzymatic (e.g., superoxide dismutase and catalase) and non-enzymatic (e.g., ascorbate) antioxidants to scavenge reactive oxygen species	Mitigates oxidative damage to membranes, proteins, and DNA	Requires complex coordination of multiple pathways; effectiveness varies with stress intensity and duration	[[55], [56], [57]]
Plant-microbe interactions	General rhizosphere microbiome engineering	Inoculation with plant growth-promoting rhizobacteria (PGPR) or arbuscular mycorrhizal fungi (AMF) from the rhizosphere	Multi-mechanistic (phytohormones and nutrient solubilization); can be applied to existing cultivars	Efficacy is highly variable depending on soil type, climate, and resident microbiota; microbes are vulnerable to desiccation in the rhizosphere	[8,58]
	Rhizosheath microbiome harnessing (focus of this review)	Utilizing microbes specifically adapted to the hydrated, stable rhizosheath microenvironment	Microbes are naturally protected from desiccation; stronger and more stable plant-microbe associations under drought	Limited field-scale validation; complex interactions within the rhizosheath community are not fully understood	[59,60]
Agronomic practices	Deficit irrigation & water harvesting	Applying water below full crop evapotranspiration needs or collecting runoff to supplement irrigation	Significant water savings; improves water use efficiency (WUE)	Requires precise scheduling; risk of yield reduction if timing/amount is incorrect	[[61], [62], [63]]
	Conservation agriculture	Use of no-till/minimum tillage, residue retention (mulching), and cover crops to improve soil health and water retention	Enhances soil organic matter and infiltration; reduces evaporation	Requires changes in farm management; residue retention can harbor pests or diseases	[[64], [65], [66]]
	Use of superabsorbent polymers (hydrogels)	Applying water-absorbing materials to the soil to increase water-holding capacity	Can significantly increase soil moisture availability around roots	Can be expensive for large-scale application; environmental persistence and long-term effects on soil are concerns	[[67], [68], [69]]

## 3 The rhizosheath: A drought-adapted microenvironment and its specialized microbiome

The inherent limitations of conventional drought mitigation strategies and the vulnerability of the general rhizosphere to water scarcity necessitate a focus on more resilient ecological niches. The rhizosheath represents precisely such a niche. In the following section, we integrate information on the physical formation and properties of the rhizosheath with a discussion of the assembly and characteristics of its resident microbiota to explain why this root–soil–microbe continuum is uniquely suited to enhance plant drought tolerance.

### 3.1 Formation and structure: creating a sanctuary at the root–soil interface

The rhizosheath is a specialized structure consisting of soil particles tightly bound to root systems by a matrix of root-derived mucilage, exudates, and microbial biofilms. Its formation is a dynamic process influenced by plant genetics (e.g., root hair density and length), soil properties (e.g., texture and organic matter), and environmental cues [24]. Drought conditions can stimulate its development by increasing mucilage secretion and root hair growth, thereby enhancing soil aggregation. The cohesive structure of the rhizosheath is fundamental to its function, as it minimizes air gaps between roots and soil, creating a critical bridge for resource transport [24,70,71].

### 3.2 A functional “mini-oasis”: how structure dictates drought resilience

**Fig. 2:**
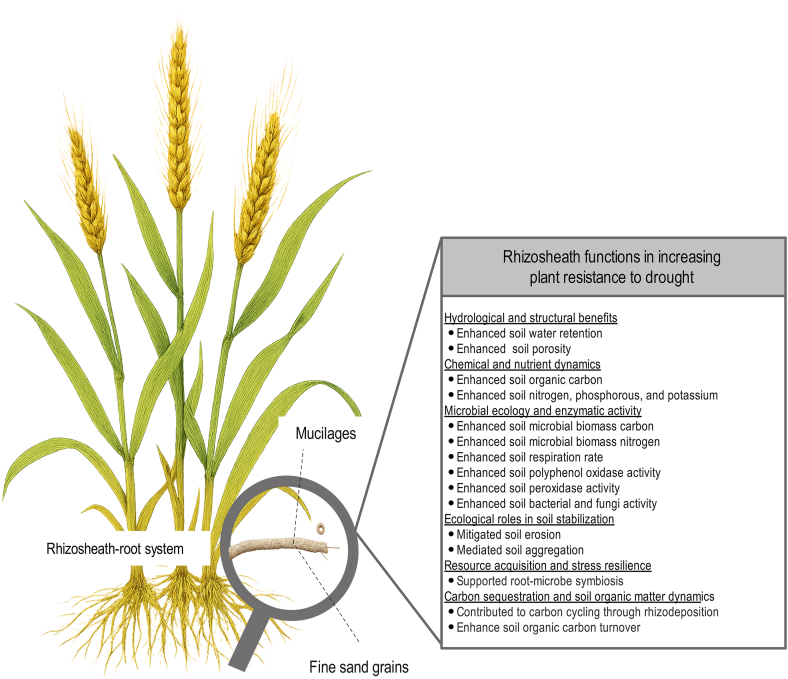
The rhizosheath and its functions in promoting plant drought resistance.

The physical architecture of the rhizosheath provides significant functional advantages under water-deficit conditions (Fig. 2). The mucilaginous matrix functions as a hydrogel, greatly increasing water-holding capacity: rhizosheath soil can retain over 60 % more moisture than the surrounding bulk soil, creating a hydrated sanctuary for both roots and associated microbes [27]. In addition, strong interactions at the root–soil–microbe interface lead to nutrient enrichment, with rhizosheath soils typically containing significantly higher levels of organic carbon, nitrogen, and soluble phosphorus, enhancing nutrient availability at the root surface [72,73]. The aggregated structure of the rhizosheath also protects roots from mechanical damage, erosion, and extreme temperature fluctuations, which are common in dryland environments [74].

### 3.3 Microbial recruitment and diversity: Selecting for a drought-tolerant consortium

**Fig. 3:**
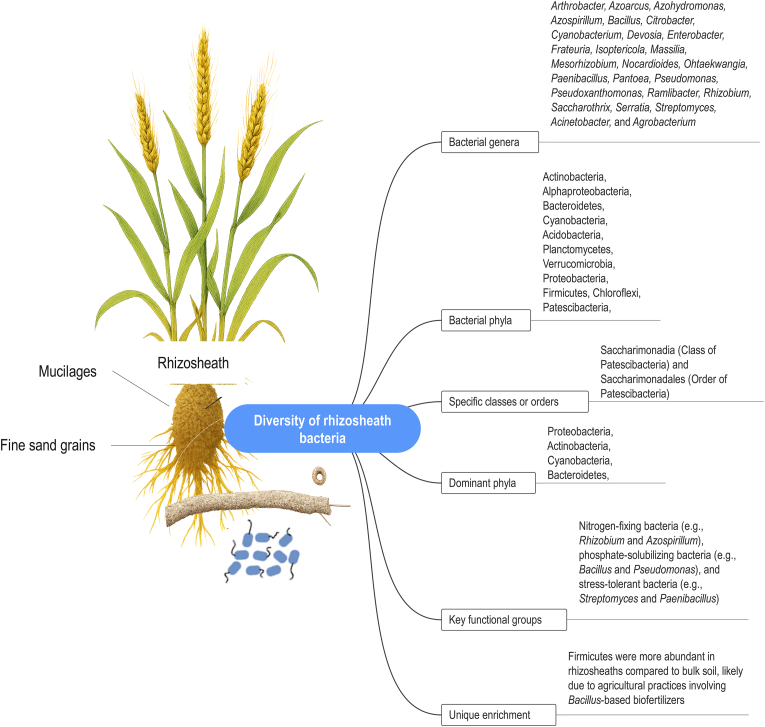
Bacterial genera, phyla, and specific classes/orders reported in the rhizosheath.

The unique physicochemical conditions of the rhizosheath—stable moisture, high nutrient availability, and a protected environment—act as a powerful filter, selectively enriching a specialized microbial community. This is not a random assemblage but a plant-mediated recruitment of drought-adapted taxa. Key bacterial phyla commonly enriched in the rhizosheath include Proteobacteria, Actinobacteria, Firmicutes, and Bacteroidetes, with notable genera such as *Bacillus*, *Pseudomonas*, *Azospirillum*, *Rhizobium*, and *Arthrobacter* [73,75] (Fig. 3). The composition of this microbiome is not static; it varies with plant species, soil type, and agricultural management practices, demonstrating a high degree of context dependency. For example, desert plants such as *Stipagrostis pennata* enrich for nitrogen-fixing genera such as *Mesorhizobium* and *Azospirillum* [76], and wheat (*Triticum aestivum* L.) selects for distinct phosphate-solubilizing communities under different crop rotations [73].

### 3.4 How microbes reinforce rhizosheath structure

**Fig. 4:**
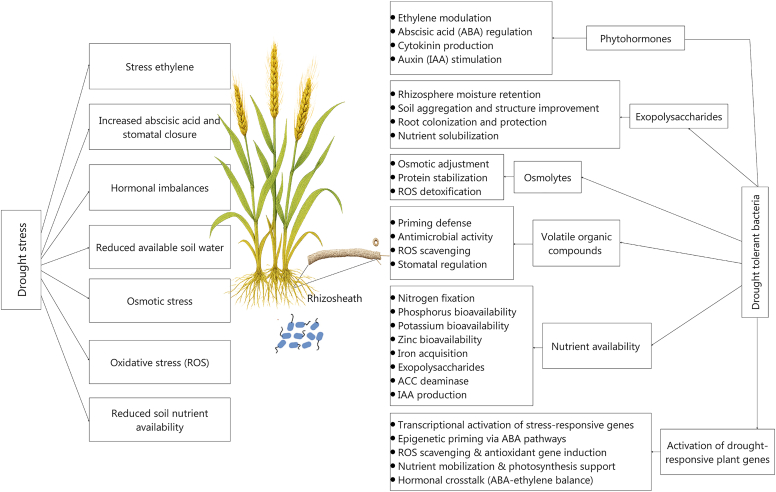
Effects of drought stress on plants and mechanisms by which bacteria mitigate these effects. Under drought conditions, plants and microbes release volatile organic compounds (VOCs), low-molecular-weight molecules that serve as signals to prime plants for faster stress responses. VOCs such as terpenoids and aldehydes exhibit antimicrobial properties, inhibit pathogen growth, and reduce oxidative stress by enhancing antioxidant enzyme activity to scavenge reactive oxygen species (ROS). VOCs such as ethylene and methyl salicylate regulate stomatal closure, promoting water use efficiency. Plants also synthesize or accumulate osmolytes—small, highly soluble molecules such as proline and glycine betaine—that balance intracellular osmotic potential, protect enzymes and membranes from damage, and act as antioxidants to mitigate oxidative damage. Bacterial exopolysaccharides (EPSs), high-molecular-weight carbohydrate polymers secreted by plant-associated bacteria, enhance drought tolerance by retaining rhizosphere moisture through hydrophilic biofilms, improving soil aggregation, and facilitating root colonization and protection. EPSs can also solubilize nutrients such as phosphorus and iron, ensuring sustained metabolic activity under water-limited conditions. Bacteria further support plants by modulating phytohormone biosynthesis and signaling. Bacterial abscisic acid (ABA) production can promote stomatal closure, and bacterial 1-aminocyclopropane-1-carboxylic acid (ACC) deaminase activity can reduce ethylene levels to delay senescence and promote root growth. Cytokinin production counteracts ABA-induced stomatal closure, and auxin (IAA) secretion stimulates root elongation and root hair formation, thus enhancing water and nutrient absorption. Bacteria can improve nutrient availability through phosphorus mobilization, siderophore-mediated iron chelation, nitrogen fixation, and potassium mobilization. Collectively, these mechanisms enable plants to maintain physiological resilience and productivity under drought stress. Bacteria can also enhance drought tolerance by activating stress-responsive genes, inducing epigenetic priming, scavenging ROS, and supporting photosynthesis through nutrient mobilization and hormonal crosstalk, promoting plant survival in water-limited environments.

The relationship between the rhizosheath and its microbiome is reciprocal. The structure not only provides a habitat for microbes but also relies on their active contributions for its formation and stability. For example, the EPSs produced by bacteria such as *Bacillus* and *Pseudomonas* serve as binding agents, strengthening soil aggregates and enhancing water retention [77,78], and bacterially produced auxins (e.g., IAA) stimulate root hair development, which is crucial for rhizosheath formation [79,80]. Bacteria with 1-aminocyclopropane-1-carboxylate (ACC) deaminase activity help regulate plant ethylene levels, reducing stress-induced ethylene that can suppress root growth during drought conditions, thereby supporting maintenance of the rhizosheath [81,82]. In summary, the rhizosheath is not merely a physical attachment of soil to roots but a dynamically formed, functionally integrated ecosystem. Its structure creates a preserved, hydrated niche that selectively hosts a consortium of beneficial, drought-adapted microbes. In return, these microbes reinforce the very structure that shelters them and directly modulate plant physiology. This tight synergy among the plant, its soil sheath, and the enclosed microbiome makes the rhizosheath a far more promising target for enhancing drought resilience than the more transient and vulnerable rhizosphere. The following sections examine the specific mechanisms (Fig. 4) through which this specialized microbiome promotes plant drought tolerance.

## 4 Mechanisms of drought-stress alleviation by bacteria

### 4.1 Enhancement of water uptake

Root system architecture, which involves root system topology, distribution, and dimensions, is critical for plant drought adaptation [83,84]. Root architectural plasticity enables plants to adjust to soil conditions, optimizing water uptake under drought [85,86]. Key drought-resilient traits include deeper roots and increased numbers of small-diameter roots, which can enhance soil exploration and hydraulic conductance [3,87]. Such traits are correlated with drought resistance in various crops [[88], [89], [90], [91]]. Smaller roots can increase the surface area for water absorption, while deep roots can access subsoil moisture [3,92]. Thus, prolific, deep-rooted systems improve drought tolerance by maximizing water extraction [93,94]. Drought-tolerant beneficial bacteria can enhance root growth and modify root architecture [4,29]. Bacterially induced changes, such as increased root surface area, improve water/nutrient uptake and overall plant resilience under drought [[95], [96], [97], [98]]. For example, maize (*Zea mays* L.) seedlings inoculated with *Alcaligenes faecalis* AF3 exhibited 10 % longer roots under drought compared with controls, which enhanced their water absorption and stress tolerance [99]. Similarly, wheat treated with *Bacillus thuringiensis* AZP2 developed longer root hairs and denser lateral roots, particularly under drought, likely improving drought adaptation [95]. Jabborova et al. [100] co-inoculated soybean (*Glycine max* (L.) Merr.) with N_2_-fixing bacteria and beneficial rhizobacteria under water-deficit conditions and observed significant improvements in root and shoot growth, nodulation, and nutrient uptake. Specifically, co-inoculation with *Bradyrhizobium japonicum* USDA110 and *Pseudomonas putida* NUU8 enhanced root length by 56 % and root dry weight by 47 % under drought stress. Although PGPB-mediated root modifications have been shown to enhance drought tolerance, further research is needed to: (1) clarify links between altered root traits and drought resilience, (2) model root–soil interactions, and (3) identify optimal root traits for breeding of drought-resistant crops [4,101].

EPSs are high-molecular-weight polymers secreted by rhizobacteria that are composed of nucleic acids, polysaccharides, proteins, and lipids [102,103] and play critical roles in biofilm formation, microbial aggregation, and stress tolerance. EPS-producing bacteria such as *Pseudomonas, Bacillus, Azospirillum,* and *Rhizobium* have been shown to enhance plant tolerance of water deficit by improving water uptake and retention [77,78,99]. EPSs act as hygroscopic agents, maintaining soil moisture by forming hydrogels around roots and enhancing soil aggregation (both macro- and micro-aggregates), thus improving water infiltration and root adhesion. Under drought stress, bacteria secrete EPSs, which form protective rhizosheaths around roots, reducing water loss and maintaining soil moisture by binding soil particles into aggregates [78,104,105]. This aggregation enhances soil structure and increases water-holding capacity and nutrient availability, particularly of phosphorus. By promoting root–soil adhesion and biofilm formation, EPS-producing bacteria increase root access to moisture and nutrients [77].

### 4.2 Modulation of phytohormone levels

Plant adaptations to drought are coordinated by a complex network of phytohormones, and some rhizosheath bacteria can actively modulate this network to the plant's advantage. Rhizosheath microbes can directly influence hormonal homeostasis to enhance drought resilience through three primary strategies: ethylene reduction, auxin-mediated root architectural changes, and stress-signaling cross-talk. Drought triggers production of the ethylene precursor ACC in plants, which can lead to growth-inhibiting levels of ethylene. Rhizosheath bacteria that produce ACC deaminase (common in genera such as *Pseudomonas*, *Bacillus*, and *Variovorax*) can hydrolyze plant-sourced ACC, thus reducing ethylene levels and mitigating their inhibitory effects on root growth [106,107]. For instance, enrichment of ACC deaminase–positive *Enterobacter* strains in drought-tolerant rice rhizosheaths was directly linked to the maintenance of root elongation under stress, a mechanism that ensures root system function and rhizosheath integrity when water is scarce. In addition, many rhizosheath bacteria actively synthesize phytohormones to stimulate growth. The production of auxins (e.g., IAA) by bacteria such as *Bacillus cereus* and *Phyllobacterium brassicacearum* directly promotes root hair development and lateral root formation [79,109], which are fundamental to rhizosheath formation and expand the root surface area for water foraging. This bacterially induced manipulation of root architecture is an important drought avoidance strategy. Rhizosheath bacteria can also influence the broader hormonal signaling landscape to fine-tune plant stress responses. Inoculation with strains of *Azospirillum* and *Bacillus* has been shown to increase abscisic acid (ABA) levels, leading to stomatal closure and reduced water loss [110,111]. This bacterial priming of the ABA pathway induces a state of readiness, enhancing osmotic stress tolerance. Similarly, bacteria can enhance plant growth under drought through effects on gibberellin (GA) and cytokinin (CK) pathways, either through direct production or by stimulating plant biosynthesis, as reported for *P. putida* H-2-3 [112] and *Burkholderia phytofirmans* [113]. The interactions between different hormonal pathways—where cytokinins, for example, can modulate ABA responses [114]—highlight the complex, systems-level hormonal recalibration orchestrated by the rhizosheath microbiome. By simultaneously lowering levels of inhibitory ethylene, increasing levels of growth-stimulating auxins, and priming stress-signaling pathways like that of ABA, rhizosheath microbes can shift the plant's hormonal balance from a stress-survival mode to a stress-resilience growth mode, thus enhancing drought tolerance.

### 4.3 Stress-tolerance compounds

Under drought stress, bacteria release low molecular weight (<300 Da) volatile organic compounds (VOCs), including ketones, aldehydes, alcohols, and sulfur compounds, which can increase crop growth and stress tolerance [115,116]. These VOCs can act without physical contact between the bacterium and the plant, inducing systemic resistance and stimulating growth via hormonal pathways (e.g., auxin and GAs) and stomatal control [117,118]. Over 300 microbial species, including members of *Bacillus*, *Pseudomonas*, and *Serratia*, produce diverse VOCs documented in databases [119]. For example, *Bacillus megaterium* XTBG34 emits 2-pentylfuran, which stimulates *Arabidopsis* growth [120], and *Pseudomonas fluorescens* SS101 releases 2-butanone and related compounds, which enhance tobacco growth and stress resilience [121]. Under water-deficit conditions, priming wheat seedlings with the *B. thuringiensis* strain AZP2 enhanced plant biomass by up to 78 % and increased survival rates fivefold. This improvement was associated with increased net carbon assimilation and reduced emission of stress-related volatiles, which contributed to enhanced drought tolerance [95]. *Pseudomonas chlororaphis* 06 produces 2R,3R-butanediol, which promotes stomatal closure to reduce water loss and activates salicylic acid–mediated water-deficit resistance in *Arabidopsis* [28,122]. Bacterially derived VOCs have also been shown to regulate microbial biofilms (e.g., acetic acid from *Bacillus subtilis* 3610) and ion transporters, improving photosynthesis and biomass under drought [123,124].

Osmotic adjustment, an important drought adaptation mechanism in plants, involves the active accumulation of compatible solutes (e.g., proline, glycine betaine, and sugars) to maintain turgor, stabilize cellular structures, and mitigate oxidative damage under water deficit [83,125,126]. By lowering cellular water potential without reducing water content, osmotic adjustment preserves membrane integrity and protein function [127,128]. Proline, a key osmolyte, accumulates markedly during drought, serving both to preserve osmotic balance and to provide cellular protection by scavenging free radicals and stabilizing proteins/membranes [[129], [130], [131]]. Increased proline levels are correlated with enhanced drought tolerance in plants such as pea, rice, chickpea, and soybean [4].

Application of beneficial bacteria can enhance proline accumulation in various plant species, improving their drought tolerance [4,99,[132], [133], [134]]. For instance, treatment of cucumber (*Cucumis sativus* L.) with *B. subtilis* SM21*, B. cereus* AR156*,* and *Serratia* sp*.* XY21 increased leaf proline levels 3–4-fold, increasing plant tolerance to water deficit [135]. Similarly, PGPB-treated maize had higher levels of free amino acids and soluble sugars, as well as proline, further enhancing drought resilience [4,29,104]. These findings underscore the role of PGPBs in increasing the content of plant osmoprotectants to combat water stress.

### 4.4 Antioxidant defense activation

Drought stress causes excess production of reactive oxygen species (ROS) such as H_2_O_2_, O_2_^−^, and HO·, which disrupt metabolism by oxidizing lipids, proteins, and DNA, leading to cell death [126,136,137]. To counteract ROS, plants deploy antioxidant enzymes (e.g., glutathione reductase, superoxide dismutase, peroxidase, catalase, and ascorbate peroxidase) and non-enzymatic antioxidant systems, which act synergistically to reduce oxidative damage [138,139]. Enhanced activity of these scavenging enzymes is correlated with drought tolerance, and robust antioxidant induction has been shown to reduce ROS accumulation [140,141]. Drought-resistant species thus prioritize efficient ROS detoxification through upregulation of antioxidant pathways [83,142]. The positive effects of PGPBs on plant drought tolerance is also linked to enhanced activity of antioxidant enzymes (e.g., catalase, ascorbate peroxidase), which mitigate oxidative damage by reducing ROS levels [4]. For instance, potato crops treated with *Bacillus firmus* 40 and *Bacillus pumilus* DH-11 showed increased ascorbate peroxidase and catalase activity, with catalase levels 1.8-fold higher under water deficit compared with controls [143]. Similar trends were observed with *B. subtilis* EPB and *P. fluorescens* Pf1 in green gram (*Vigna radiata* L.) [144], cucumber [135], maize [132], and wheat [145]. Under drought-stress conditions, treatment of basil (*Ocimum basilicum* L.) with a microbial consortium containing *Bacillus lentus*, *Azospirillum brasilense*, and *Pseudomonas* sp. significantly enhanced the activity of antioxidant enzymes such as ascorbate peroxidase and glutathione peroxidase [146], improving plant drought tolerance. These findings highlight ROS-scavenging enzymes as central to PGPB-induced drought resilience. Key unresolved questions include: (1) the strain- or plant-specificity of enzyme induction, (2) which specific antioxidant enzymes are preferentially induced by different PGPB strains, (3) correlations between enzyme levels and drought duration, (4) crop-specific variation in antioxidant responses, (5) identification of novel PGPB-induced enzymes, and (6) translation of increased enzyme activity into overall plant fitness [4].

### 4.5 Enhancement of nutrient availability and uptake

Drought significantly disrupts soil nutrient dynamics by reducing water availability, thereby limiting the diffusion and mobility of essential nutrients like nitrogen, phosphorus, potassium, and micronutrients in the soil [[147], [148], [149], [150]]. Dry conditions also impair soil structure and microbial activity, slowing the processes of organic matter decomposition and mineralization that are critical for release of plant-available nutrients. As soil moisture declines, root function is compromised, further restricting nutrient uptake and leading to deficiencies that can stunt plant growth [106,151,152]. Certain bacteria can play a vital role in mitigating these challenges. They enhance nutrient availability by mobilizing phosphates, potassium, and zinc; fixing N_2_; and producing organic acids or siderophores that chelate minerals (e.g., Fe), increasing their accessibility to plants. In addition, bacteria like *Azospirillum* and *Pseudomonas* secrete exopolysaccharides that improve soil moisture retention and root adhesion, while others produce ACC deaminase, which decreases ethylene levels, promoting root elongation and nutrient foraging [77,106,153]. By maintaining nutrient cycling and fostering symbiotic relationships with plants, these bacteria can bolster resilience to drought, ensuring sustained nutrient uptake and crop productivity under water-limited conditions.

### 4.6 Modulation of drought-responsive genes in crops

Plants respond to drought by altering the expression of a wide range of genes involved in various physiological and biochemical pathways [154]. Bacteria can also enhance water-deficit tolerance by modulating the expression of drought-responsive genes [28,29,155]. For instance, inoculation of *Arabidopsis thaliana* with *Paenibacillus polymyxa* increased the transcription of *Early Responsive to Dehydration 15* (*ERD15*) genes, thereby alleviating water-deficit stress [28,156]. In rice (*Oryza sativa* L.), inoculation with *P. fluorescens* upregulated transcription factors such as COC1 and bZIP1, as well as Hsp20 proteins, contributing to water-deficit tolerance [157]. Similarly, in pepper (*Capsicum annuum*), *Bacillus licheniformis* inoculation enhanced the expression of genes associated with water-deficit tolerance, such as *sHSP*, *CaDHN*, *Ca-PR-10*, and *VA* [158]. In addition, *P. chlororaphis* O6 inoculation in *A. thaliana* roots increased the expression of *LEA* and *dehydrin* genes, significantly enhancing drought tolerance [159]. Gagné-Bourque et al. [160] demonstrated that colonization of *Brachypodium distachyon* by *B. subtilis* B26 led to upregulation of genes such as *LEA-14-A-like*, *DREB2B-like*, and *DHN3-like*; these genes are associated with water-deficit tolerance, and their expression was linked to improved drought resilience. The root endophytic bacterium *Pseudomonas argentinensis* strain SA190 enhanced water-deficit tolerance in *Arabidopsis* by modulating gene expression through the ABA pathway, a mechanism that involved epigenetic priming of drought-responsive genes [161]. A consortium of beneficial rhizobacterial strains, including *B. subtilis* SM21, *Serratia* sp. XY21, and *B. cereus* AR156, induced water-deficit tolerance in cucumber by enhancing the expression of genes encoding cytosolic ascorbate peroxidase and ribulose-1,5-bisphosphate carboxylase/oxygenase subunits, which are crucial for maintaining photosynthetic efficiency under water deficit [135]. Together, these examples illustrate how bacteria can modulate gene expression to improve drought resistance in plants.

## 5 Ecological significance of dominant bacterial genera in the rhizosheath

Specific bacterial genera are consistently observed across diverse rhizosheath systems, providing strong evidence for functional selection pressures. The prevalence of taxa such as *Pseudomonas*, *Bacillus*, *Azospirillum*, *Rhizobium*, and *Arthrobacter* reveals a pattern of ecological filtering for traits that enable survival in this unique microenvironment and that provide benefits to the host plant under drought stress [73,176]. In the following section, we discuss the ecological significance of these dominant groups (Table 3) and examine what their dominance tells us about the rhizosheath as a functional unit.

**Table 3:** Plant species, associated rhizosheath bacteria, and their plant growth-promoting traits.

Plant species	Rhizosheath bacteria	Plant growth-promoting activities	Reference
Wheat (*Triticum aestivum* L.)	*Bacillus, Arthrobacter, Azospirillum, Paenibacillus*, *Pseudomonas*, *Gordonia, Cyanobacterium*, and *Rhizobium*	Nitrogen fixation, phosphate solubilization, and IAA production	[73]
Rice (*Oryza sativa* L.)	*Massilia, Nocardioides, Frateuria,* and *Angustibacter*	IAA production, potassium uptake enhancement, IAA, and salicylic acid production	[162,163]
*Stipagrostis pennata*	*Mesorhizobium, Azohydromonas, Azospirillum, Ohtaekwangia, Saccharothrix, Cytophagaceae, Planctomycetaceae,* and *Rhodospirillaceae*	Nitrogen fixation, phosphorus mobilization, phytohormone production, and biocontrol activities	[76,164]
Switchgrass (*Panicum virgatum* L.)	*Citrobacter, Acinetobacter, Pseudomonas*, *Bacillus*, and *Streptomyces*	Exopolysaccharide production, IAA production, ACC deaminase activity, phosphate, and potassium solubilization	[165]
*Kengyilia hirsuta*	*Arthrobacter*	IAA production and root elongation stimulation	[166]
*Stipagrostis ciliata*	*Bacillus, Paenibacillus*, *Nocardioides*, *Isoptericola*, *Streptomyces*, *Acinetobacter*, *Ramlibacter*, and *Pseudoxanthomonas*	Phosphate solubilization, nitrogen fixation, EPS production, antibiotic production, siderophore production, hydrolytic enzyme production, organic matter degradation, stress alleviation through ACC deaminase activity, and biocontrol activities	[167]
Mustard (*Brassica nigra* L.), Phacelia (*Phacelia tanacetifolia* L.), and Buckwheat (*Fagopyrum esculentum* L.)	*Pseudomonas*, *Bacillus*, and *Rhizobium*	Organic phosphorus solubilization (alkaline phosphomonoesterase production), nutrient cycling, and improved phosphorus nutrition for subsequent crops	[168]
Various plants	*Enterobacter aerogenes*	ACC deaminase production, ethylene reduction, and rhizosheath formation promotion	[82]

### 5.1 A convergence of stress-adapted life history strategies

The dominance of genera known for their ability to form spores (*Bacillus*) or produce abundant EPSs (*Pseudomonas* and *Azospirillum*) points to ecological filtering for life history strategies suited to fluctuating water availability [7,78,169]. Such traits can be viewed as pre-adaptations that enable these bacteria to withstand the periodic desiccation that occurs at the soil–root interface, even within the protected rhizosheath [166,170]. Their prevalence indicates that the rhizosheath, while more hydrated than the bulk soil, still imposes a strong selective pressure for desiccation tolerance, favoring microbes that can rapidly transition between active and dormant states.

### 5.2 Evidence of a mutualistic feedback loop

Enrichment of PGPBs with specific functions in the rhizosheath provides strong evidence of a mutually beneficial feedback loop. Plants likely recruit these microbes through their root exudate profiles, and in return, the microbes offer invaluable services, particularly under drought conditions. The prevalence of genera such as *Azospirillum*, *Rhizobium*, and *Mesorhizobium* highlights the critical importance of nitrogen acquisition during stress, especially when nutrient diffusion is limited [73,164]. Similarly, the presence of phosphate-solubilizing bacteria like *Bacillus* and *Pseudomonas* underscores the necessity of unlocking immobilized phosphorus in dry soils [[167], [168], [169]]. This suggests that the plant microbiome is functionally structured to compensate for nutrient deficiencies induced by drought. In addition, the prevalence of strong EPS producers, such as *Pseudomonas* and *Bacillus*, indicates that bacteria that contribute to the physical integrity of the rhizosheath are rewarded with a stable habitat [82,171]. This scenario offers a fascinating example of niche construction, in which microbes reinforce the very environment that selects for their presence.

### 5.3 Plant-specific recruitment and the “core” vs. “context-dependent” microbiome

Variations in the composition of the rhizosheath community—for example, *Massilia* and *Frateuria* in rice [162] versus *Streptomyces* and *Pseudoxanthomonas* in desert grasses [167]—are not noise but signals. They suggest that different plant species can selectively recruit distinct bacterial cohorts to solve similar problems (i.e., drought stress), a concept known as functional redundancy [172]. This has profound implications for application of PGPBs: it suggests that although a universal “magic bullet” strain may be elusive, a core functional profile (e.g., EPS production, ACC deaminase, and nutrient solubilization) may be conserved. The specific bacterial taxa that fulfill these functions are context dependent, shaped by plant genotype and soil history [73,165]. Therefore, the goal shifts from finding universally dominant taxa to understanding the core functions that need to be engineered or enhanced within a given agricultural context.

### 5.4 Implications for synthetic community (SynCom) design

Understanding the reasons for the dominance of specific genera provides a blueprint for designing effective synthetic microbial communities (SynComs). The principles derived from rhizosheath studies suggest that optimal SynComs should include structure-builders, such as EPS producers to stabilize the niche; nutrient providers, including nitrogen-fixers and phosphate-solubilizers to increase nutrient accessibility under drought; plant growth modulators, i.e., strains that produce ACC deaminase and phytohormones to maintain root growth under stress; and stress-tolerant pioneers, i.e., genera known for their resilience to osmotic stress. The consistent presence of these functional groups in rhizosheaths from various environments suggests their synergistic importance and provides a data-driven foundation for the development of microbial inoculants [7].

## 6 Effects of rhizosheath bacterial inoculation on crop growth

Inoculation with rhizosheath-associated bacteria has been shown to enhance plant growth and stress resilience in multiple systems. For instance, inoculation of wheat with *Azospirillum* sp. WS-1 or *Bacillus* sp. T-34 in wheat–cotton (*Gossypium herbaceum* L.) and wheat–rice rotations increased root length, shoot/root biomass, and rhizosheath dry weight, with the wheat–cotton system showing greater improvements. Inoculated plants also exhibited higher relative abundance of beneficial bacterial genera (0.78–0.80 %) compared with non-inoculated controls (0.52 %), together with increased rhizosheath concentrations of malic acid (24.8 μg g^−1^ in wheat–cotton) and altered concentrations of sugars, which influence microbial recruitment [73]. Drought stress also shapes the rhizosheath microbiome, as seen in switchgrass (*Panicum virgatum* L.), where *Acinetobacter* and *Citrobacter* were enriched in drought-tolerant ecotypes, improving moisture retention and nutrient uptake [165]. Similarly, the presence of *Pseudomonas*, *Bacillus*, and *Rhizobium* triggered immune pathways in rice (e.g., FLS2 and EFR) and upregulated calcium/MAPK signaling genes, enhancing drought resilience in tolerant varieties such as Gaoshan 1 [173]. Brassicaceae and Fabaceae species (e.g., *Brassica carinata* L. and *Vicia faba* L.) hosted carboxylate-responsive bacteria such as *Pseudomonas* and *Rhizobium*, which mobilized phosphorus via root exudates (e.g., malate) [174]. The bacterium *Pantoea agglomerans* from the desert grass *S. pennata* promoted wheat growth under drought, increasing root volume (by 45 %), shoot nitrogen concentration (by 18 %), and antioxidant enzyme activity (e.g., superoxide dismutase) while reducing proline levels [169]. The rhizosheaths of another desert plant, the pioneer grass *Kengyilia hirsuta*, were enriched in *Massilia* (implicated in soil aggregation and nutrient solubilization) and *Arthrobacter* (associated with IAA production and root elongation), which may promote its survival in nutrient-poor sandy soils [166]. These findings suggest that the enrichment of specific bacteria in the rhizosheath may enhance plant resilience to drought stress by improving nutrient acquisition (e.g., phosphate mineralization via *phoD*) and mitigating abiotic stress through the production of exopolysaccharides and ACC deaminase and the activation of plant immune pathways. These interactions are shaped by plant-specific exudates (e.g., carboxylates and sugars) [73] and environmental conditions.

## 7 Roles of bacterial communities in rhizosheath formation

Rhizosheath formation is influenced by plant root traits (e.g., root hair length and density), soil properties (texture, organic matter, and wet–dry cycles), and root exudates, particularly mucilage—a polysaccharide-rich secretion that binds soil particles via hydrogen bonding, enhances water retention, and reduces root–soil air gaps [24,[175], [176], [177]]. Although root hairs are strongly correlated with rhizosheath size in many plants [178,179], some angiosperms form rhizosheaths independently of root hairs, suggesting species-specific mechanisms [24]. Soil texture also plays a role, with sandy soils and frequent wet–dry cycles favoring aggregation [79,180]. The hygroscopic properties of mucilage maintain rhizosheath moisture during drought, while other exudates (organic acids, flavonoids, and phenolic compounds) recruit beneficial microbes that indirectly support soil stability [170,181].

**Fig. 5:**
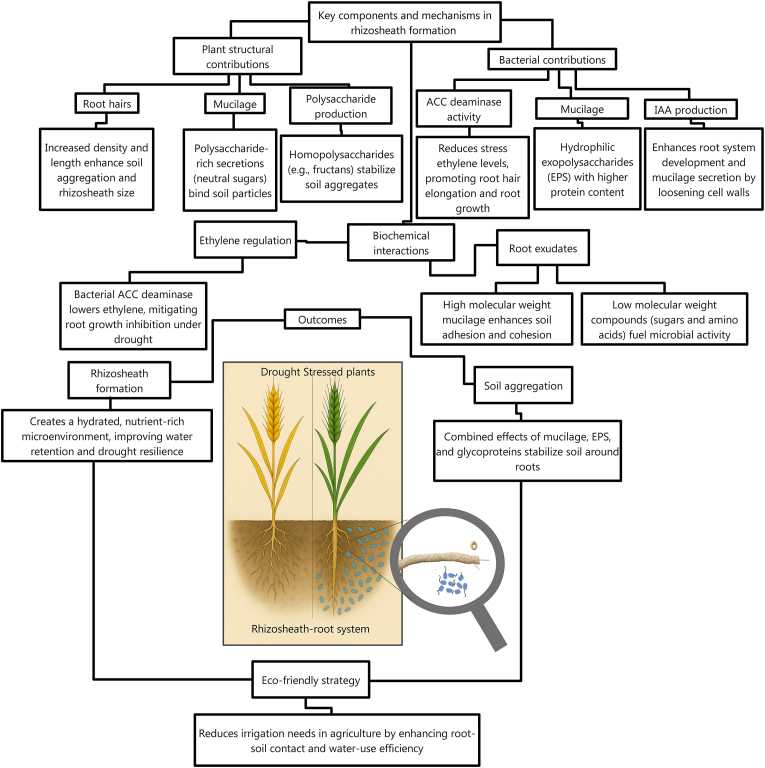
Roles of plants and bacteria in rhizosheath formation.

Bacterial communities contribute to rhizosheath formation through multiple mechanisms (Fig. 5): (i) Ethylene reduction: ACC deaminase-producing bacteria (e.g., *Enterobacter aerogenes* isolated from the rhizosheath of drought-tolerant rice) limit root ethylene production under drought, thus promoting root elongation and rhizosheath formation. Deletion of the *acdS* gene in *E. aerogenes* abolished this effect [81,82,182]. (ii) EPS production: EPS-producing bacteria like *Bacillus*, *Azospirillum*, *Rhizobium*, and *Mesorhizobium* enhance soil aggregation and moisture retention. Inoculation with these strains increases rhizosheath EPS content (glucose, xylose, and galactose) under drought, strengthening soil–root contact [73,171]. Desert grasses such as *Stipagrostis pungens* rely on EPS-producing rhizosheath bacteria to thrive in arid soils [171]. (iii) Phytohormone signaling: Bacteria such as *B. cereus* and *Paenibacillus polymyxa* produce auxins (e.g., IAA) that stimulate root hair growth and rhizosheath development. IAA-deficient mutants of *Chryseobacterium culicis* and *P. polymyxa* failed to enhance barley rhizosheath formation, whereas wild-type strains increased grain yield in field trials [79,80]. Similarly, auxin- and cytokinin-producing microbes can modulate root architecture, improving drought adaptation [108,183]. (iv) Nutrient mobilization: Inoculation with *Bacillus* and *Azospirillum* strains increases the concentrations of organic acids (citric and malic acids) in wheat rhizosheaths, improving rhizosheath formation and enhancing nutrient solubility and root biomass under crop rotations [73].

## 8 Future directions and practical opportunities

To translate the promising potential of rhizosheath microbiomes into tangible agricultural solutions for drought resilience, future research must address specific knowledge gaps and focus on the development of scalable application strategies. Priorities for future research and practical approaches for harnessing beneficial microbial communities are outlined below.

### 8.1 Characterization of rhizosheath mechanisms and plant–microbe specificity

Although some mechanisms such as ACC deaminase activity and EPS production are well documented, a more complete understanding of rhizosheath function is needed. Future work should therefore focus on:

• Integrated multi-omics approaches: Metagenomics, transcriptomics, metabolomics, and proteomics can be used to examine plant–microbe signaling pathways under drought stress. These approaches can help to identify novel drought-responsive genes, metabolites, and interactions beyond currently known mechanisms.
• Decoding specificity: Research should focus on identifying the factors that govern strain–plant specificity. Key questions include: How do plant genotypes and root exudate profiles drive the recruitment of specific beneficial bacteria? How do soil type, historical management, and drought intensity influence the effectiveness of inoculated strains?
• Understudied mechanisms: The roles of microbial VOCs and quorum sensing in rhizosheath formation and drought tolerance remain largely unexplored.

### 8.2 Development of effective microbial inoculants and consortia

Moving from single-strain inoculants to more robust and effective solutions is crucial.

• Design of SynComs: Development of consortia that include taxa with complementary traits (e.g., EPS production + ACC deaminase + nutrient solubilization) may create synergistic benefits for plants. This approach could enhance plant resilience and rhizosheath function compared with single-strain applications.
• Advanced formulation and delivery systems: Innovative carrier materials (e.g., biochar, clay composites, and hydrogels) could protect bacteria from desiccation during storage and application on dry soils. Strategies such as seed coating with rhizosheath bacteria or integration of such bacteria into irrigation systems (“microbe-enhanced hydrology”) require optimization for field reliability.

### 8.3 Translational research

Translating laboratory findings into practical agricultural applications is the ultimate goal. Key strategies for harnessing rhizosheath microbiomes include:

• Field-scale validation and long-term studies: Field trials to assess inoculant persistence, soil health effects, and yield stability in diverse agroecosystems under variable climates should be prioritized. Long-term studies will be essential for evaluating ecological impacts and durability.
• Precision microbiome management: Predictive algorithms could be developed to recommend optimal, context-specific microbial consortia on the basis of soil, climate, and plant data. This precision approach could be combined with soil moisture sensors for real-time irrigation management, thus maximizing water-use efficiency.
• Harnessing indigenous microbiomes: Native, drought-adapted rhizosheath bacteria (e.g., from desert plants such as *Stipagrostis*) could offer ecological advantages over introduced strains, promoting adaptation and preserving local biodiversity.
• Plant-based strategies: Crop breeding or genetic engineering could be used to enhance traits that promote beneficial rhizosheath formation, such as root hair density and mucilage composition, thereby naturally enriching the soil with beneficial microbes.

### 8.4 Advancing analytical and ecological understanding

Technological advances and ecological insights will underpin future progress.

• Advanced imaging and sensing: Technologies such as X-ray computed tomography and hyperspectral imaging could be used to non-destructively quantify rhizosheath architecture, soil aggregation, and root–microbe dynamics *in situ.*
• Ecological and evolutionary dynamics: Future work should investigate how repeated drought cycles alter rhizosheath microbial communities and their resilience. Understanding evolutionary adaptations, including the potential for horizontal gene transfer among drought-tolerant microbial taxa, could inform the design of durable inoculants.
• Risk assessment: Potential unintended consequences, such as enrichment of undesirable microbial taxa or disruption of native soil ecosystems, should be proactively assessed to ensure the sustainability of microbiome-based interventions.

These research directions and application strategies can accelerate the development of rhizosheath microbiome–based solutions, paving the way for more resilient and sustainable agricultural systems in the face of increasing water scarcity.

## 9 Conclusions

Drought stress poses a formidable threat to global agriculture, exacerbating food insecurity and destabilizing ecosystems. In this context, rhizosheath-associated bacteria have emerged as an important biological resource for enhancing plant resilience to water scarcity. The rhizosheath, a unique root–soil–microbe interface, acts as a drought-adaptive “biofilm” that improves water retention, nutrient availability, and microbial activity in arid environments. Key bacterial genera such as *Pseudomonas*, *Azospirillum*, *Rhizobium,* and *Bacillus* promote drought tolerance through multiple mechanisms, including ethylene reduction, EPS-enhanced soil aggregation, phytohormone signaling, and antioxidant defense activation. Such microbes not only modulate root architecture and stress-responsive gene expression but also foster synergistic interactions with plants, enabling efficient resource acquisition and ROS mitigation under drought.

Despite these advances, critical challenges persist. The efficacy of rhizosheath bacteria varies across plant species, soil types, and environmental conditions, highlighting the need for tailored, context-specific solutions. Although lab studies have demonstrated remarkable potential, field-scale validation remains limited, particularly in diverse agroecosystems. Furthermore, the ecological and evolutionary dynamics of rhizosheath microbiomes—such as community resilience to repeated droughts or trade-offs between native and introduced strains—are poorly understood. Interdisciplinary collaboration among microbiologists, agronomists, data scientists, and policymakers will be needed to optimize microbial consortia, develop scalable bioformulations, and integrate microbiome-based strategies into climate-smart agriculture. Innovations in CRISPR-based microbial engineering, carrier technologies for inoculant delivery, and precision agriculture tools could revolutionize drought mitigation. At the same time, farmer engagement and policy frameworks must prioritize sustainable practices that harmonize microbial solutions with ecological balance. By characterizing the complexities of plant–microbe–soil interactions and fostering global partnerships, we can harness the rhizosheath microbiome to build resilient agricultural systems capable of withstanding climate extremes. In doing so, we move closer to securing food production for future generations while safeguarding the health of the planet.

## CRediT authorship contribution statement

**Hassan Etesami:** Writing – review & editing, Writing – original draft. **Amir Hosein Yadegari:** Writing – review & editing. **Umarov Otabek:** Writing – review & editing. **Bafayeva Zahro:** Writing – review & editing. **Nafetdinov Shavakatullo:** Writing – review & editing. **Avezov Tolqinjon:** Writing – review & editing.

## Declaration of competing interest

The authors declare that they have no known competing financial interests or personal relationships that could have appeared to influence the work reported in this paper.

## Data Availability

No data was used for the research described in the article.
